# Downregulating Serine Hydroxymethyltransferase 2 Deteriorates Hepatic Ischemia-Reperfusion Injury through ROS/JNK/P53 Signaling in Mice

**DOI:** 10.1155/2019/2712185

**Published:** 2019-11-18

**Authors:** Hao Wu, He Bai, Shigang Duan, Fangchao Yuan

**Affiliations:** ^1^Department of Hepatobiliary Surgery, The Second Affiliated Hospital of Chongqing Medical University, Chongqing 400000, China; ^2^Department of General Surgery Department, The First Affiliated Hospital of Xi'an Medical University, No. 48 Fenggao Street, Lianhu District, Xi'an 710000, China; ^3^Department of Hepatobiliary Surgery, The Ninth People's Hospital of Chongqing, Chongqing 400799, China

## Abstract

**Background:**

Serine hydroxymethyltransferase 2 (SHMT2) activity ensures that cells have a survival advantage in ischemic conditions and regulates redox homeostasis. In this study, we aimed to investigate the role of SHMT2 after hepatic ischemia-reperfusion (IR), which involves hypoxia, ischemic conditions, and cell apoptosis.

**Methods:**

A 70% IR model was established in C57BL/6J mice with or without SHMT2 knockdown. H&E staining, liver weight/body weight, serum alanine aminotransferase (ALT), and aspartate aminotransferase (AST) levels and cell apoptosis were tested to analyze liver damage and function. Then, the related cellular signals were probed.

**Results:**

The level of SHMT2 protein was significantly increased at 24 h and 48 h after IR (*p* < 0.001). Mice in the shSHMT2 group showed more necrotic areas and histological damage at 24 h (*p* < 0.01) after IR and higher levels of serum ALT and AST (*p* < 0.05) compared with those of mice in the scramble group. After IR for 24 h, the expression of TUNEL in the shSHMT2 group was significantly higher than that in the scramble group, as shown by histological analysis (*p* < 0.01). Mechanistically, the JNK/P53 signaling pathway was activated by IR, and knockdown of SHMT2 exacerbated hepatocyte apoptosis.

**Conclusions:**

Knockdown of SHMT2 worsens IR injury through the ROS/JNK/P53 signaling pathway. Our discovery expands the understanding of both molecular and metabolic mechanisms involved in IR. SHMT2 is a possible therapeutic target to improve the prognosis of liver transplantation (LT) and subtotal hepatectomy.

## 1. Introduction

Hepatic ischemia-reperfusion (IR) injury may lead to liver graft nonfunction and liver failure following resection and liver transplantation [1]. Hepatic IR injuries that occur during operations may impede the restoration of liver function after surgery. Previous studies have shown that hepatic IR injury is induced by metabolic acidosis, excess intracellular generation of oxygen-free radicals, and neutrophil activation [2, 3]. Thus, preventing IR is still a clinical challenge at present.

Serine hydroxymethyltransferase 2 (SHMT2) is the central enzyme that regulates the exchange between serine catabolism and single-carbon metabolism. SHMT2 plays a regulatory role in cell proliferation and redox homeostasis by regulating small molecular metabolites [4]. SHMT2 activity ensures that cells in ischemia conditions survive by limiting pyruvate kinase (PKM2) and reducing oxygen consumption [5]. SHMT2 has been verified as a necessity for maintaining redox homeostasis and cell survival under hypoxic conditions [6]. Here, we hypothesize that there might be some changes in the expression of SHMT2 under IR conditions that contain both hypoxia and ischemia. To our knowledge, few studies have investigated the expression or effect of SHMT2 in an IR model.

c-Jun NH2-terminal kinase (JNK) is a member of the mitogen-activated protein kinase (MAPK) superfamily and is induced by cytokines and environmental stress [7]. The JNK signaling pathway is related to multiple physiological processes, including cell growth, cell differentiation, and programmed death [8]. JNK can be activated by hepatic I/R injury [7, 9]. Phosphorylation and activation of JNK are induced by cytokines, including TNF-alpha and IL-1, and stresses, including radiation and oxidative stress [10, 11]. Apoptosis is the primary method of programmed cell death, through which organisms are able to maintain tissue homeostasis by removing excess or damaged cells [12]. The JNK pathway regulates cell death through the core apoptotic pathway [13]. Previous studies have verified that JNK can affect mitochondria and cause apoptosis directly. JNK is activated during warm and cold hepatic I/R injury induced by liver transplantation and is strongly induced during warm hepatic I/R injury and during cold ischemia/warm repetition injury in liver transplantation [7].

The present study examined the expression of SHMT2 in an IR mouse model and showed that impaired SHMT2 expression induced JNK activation and promotes apoptosis, exacerbating hepatic ischemia-reperfusion injury.

## 2. Methods

### 2.1. Animals

Male C57BL/6 mice (4–8 weeks old; 19–23 g) were purchased from the Experimental Animal Center of Chongqing Medical University (Chongqing, China). The mice were kept under conditions of a specific pathogen-free atmosphere and were housed at a temperature of 23°C and humidity of 60% under a 12 h light/dark cycle. Animal experiments complied with the guidelines of the China Association of Laboratory Animal Care.

### 2.2. Hepatic IR Model

A mouse model of warm hepatic ischemia followed by reperfusion was used as described previously [14]. After exploratory laparotomy, the portal vein branch on the left side of the liver was clamped with a topless blood clip, resulting in 70% hepatic ischemia. The hemostatic clip was released to open the blood return after 60 minutes of clamping. The mice were divided stochastically into four groups: sham group, in which the mice only received the open laparotomy without ischemic treatment; negative control group, in which the mice were injected with saline through tail vein before undergoing the operation; AAV8-scramble group, in which the mice were injected via the tail vein with AAV8-scramble adeno-associated virus 4 weeks before undergoing the operation; and AAV8-shSHMT2 group, in which the mice were injected via the tail vein with AAV8-ShRNA-SHMT2 adeno-associated virus 4 weeks before undergoing the operation. After the operation, the mice were euthanized at 4, 8, 16, 24, or 48 h. Then, we collected serum to be centrifuged and placed fresh liver tissue in a liquid nitrogen tank for preservation. AAV8 was purchased from Genepharma (China), and the sequence of the short hairpin scramble antisense was TGTGAGGAACTTGAGATCT, while the sequence of shSHMT2 was GGACGGGCCAGGAGAGTTTAT.

### 2.3. Isolation of Primary Hepatocytes and Cell Culture

Primary hepatocytes were extracted according to a method introduced by Edwards et al. [15]. In short, the mice were given general anesthesia, followed by laparotomy and inferior vena cava (IVC). After the buffer was injected into the inferior vena cava (Ca2+- and Mg2+-free Hanks balanced salt solution (HBSS)), the vessel became blocked below the heart. At this point, buffer 2 (Ca2+- and Mg2+-containing HBSS to a final concentration of 0.08 U/mL) was perfused, and the infusion continued for approximately 8–10 minutes, lasting a total of 18 minutes (5 mL/min). The liver was then placed in a 100 mm cell culture plate to further isolate the liver cells. Finally, a 1 mL (5 × 10^6^ hepatocytes/mL) suspension was distributed to each well of the 12-well culture plate and incubated at 37°C for later use. The cells were divided into five groups: NC group as the control group without any treatment; scramble group was transfected with the scramble adenovirus vector; Hypo + scramble group, which was transfected with scramble adenovirus vector, cultured under hypoxic conditions (95% N_2_, 5% CO_2_, 37°C) for 30 min and then incubated at 37°C with 5% CO_2_ for 24 h; Hypo + advSHMT2 group was transfected with a SHMT2-overexpressing adenovirus vector, cultured under hypoxic conditions (95% N_2_, 5% CO_2_, 37°C) for 30 min and then incubated at 37°C with 5% CO_2_ for 24 h; and Hypo + shSHMT2 group was transfected with a SHMT2-silencing adenovirus vector, cultured under hypoxic conditions (95% N_2_, 5% CO_2_, 37°C) for 30 min and then incubated at 37°C with 5% CO_2_ for 24 h.

### 2.4. Liver Histopathology

The collected tissues were fixed in a 10% neutral tissue fixing solution and embedded with paraffin after gradient dehydration with alcohol, and 5 *μ*m thick slices were fixed on the glass slides. Hematoxylin and eosin staining was used to observe tissue damage. The sections were dehydrated with different concentrations of ethanol and xylene and sealed with neutral gum, and the pathological changes were observed under an optical microscope. Then, we used Suzuki's criteria to assess the severity of IRI. Suzuki's criteria were scaled from 0 to 4.

### 2.5. Immunofluorescence

Sections were deparaffined and hydrated before antigen retrieval in 10 mM citric acid buffer. Then, the sections were incubated in 1% Triton X-100 for 15 min, and 3% hydrogen peroxide was used for 15 min to remove endogenous peroxidase activity. The sections were washed with PBS three times and incubated with primary anti-SHMT2 antibody (Abcam Inc.) at 4°C overnight. Then, the sections were washed with PBS and incubated with specific secondary antibodies labeled with tetramethylrhodamine (red) and DAPI (blue) for 30 min at room temperature. The sections were then washed with PBS and sealed with Fluor Fluoromount-G™ slide mounting medium (Southern Biotech, Birmingham, AL, USA). Images were taken using a Zeiss LSM 510 confocal microscope (magnification: 200; ZeissAG, USA).

### 2.6. TUNEL Staining

Apoptotic cells were tested using the terminal deoxyuridine 50-triphosphate gap terminal marker (TUNEL) kit mediated by terminal deoxynucleotide transferase (Rog Inc., Switzerland) as described in the manual. TUNEL-positive cells were quantified by calculating the average number of positive cells in 5 random fields in each slice.

### 2.7. Detection of Liver Function

The collected mouse blood was centrifuged at a centrifugal force of 20 g and a temperature of 4°C for 10 minutes, and the serum was collected for testing. Elevated serum alanine aminotransferase (sALT) and aspartate aminotransferase (sAST) were measured with Infinity ALT and AST liquid stable reagent (Thermo Scientific, Rockford, IL, USA).

### 2.8. Measurement of Intracellular ROS

The extracted primary liver cells were placed in McM-H2DCFDA for 25 min and incubated at 37°C for 30 minutes. Then, the cells were removed and washed with PBS 3 times. Fluorescence intensity at 488 nm and 525 nm excitation wavelengths was measured using a luminometer (Tecan, Salzburg, Austria). In this experiment, the ROS intensity in untreated cells was considered to be 100%.

### 2.9. Western Blot Analysis

The collected liver tissues were homogenized in tissue lysis buffer, each group was numbered, and a total protein concentration measurement kit was used to determine the concentrations. A 12% sodium dodecyl sulfate-polyacrylamide gel was prepared, and 40 *μ*g of protein was added per lane. Proteins of different molecular weights were separated by electrophoresis and transferred to a 0.22 *μ*m poly-vinylidene fluoride membrane. The membranes were blocked in 5% BSA for 1 h, followed by incubation with primary antibodies including SHMT2 (Abcam Inc.), p-JNK (Abcam Inc.), JNK (Abcam Inc.), p-p53 (Abcam Inc.), p53 (Abcam Inc.), TNF-*α* (Abcam Inc.), IL-1*β* (CST Inc.), cleaved-caspase-3 (Abcam Inc.), caspase-3 (Abcam Inc.), and HIF-1*α* (Abcam Inc). After primary antibody incubation, the membrane was washed 3 times with TBST, and the secondary antibody was applied at room temperature (Sigma-Aldrich, USA) for 1 h. Then, chemiluminescence reagent (Thermo Scientific, USA) was used to visualize the results. All images were analyzed using Image Lab software. GADPH was used as a control.

### 2.10. Annexin V/PI Staining Assay

Primary hepatocytes were extracted and washed twice with PBS. Then, the binding buffer was added to resuspend the cells, the membrane-linking protein V and PI were added to stain the cells, and the cells were placed in fluorescence culture at 37°C for 15 minutes. Apoptosis was evaluated and quantified using flow cytometry. Flow cytometric data were acquired using a FACSCalibur and analyzed using cell quest version 5.1 software (Bd Biosciences, Franklin Lakes, NJ, USA).

### 2.11. Statistical Analysis

The results are expressed as the mean ± standard deviation (SD), and statistical analyses were performed by using SPSS 20.0 software with bar graphs representing the means of data from all samples and error bars indicating SD. Comparisons between two groups were performed using a two-tailed *t* test, while comparisons between more than two groups were performed using a one-way analysis of variance followed by a Bonferroni post hoc test. Values of *p* < 0.05 were considered statistically significant.

## 3. Results

### 3.1. Expression of SHMT2 in Liver Tissue after Hepatic Ischemia-Reperfusion Injury

**Figure 1:**
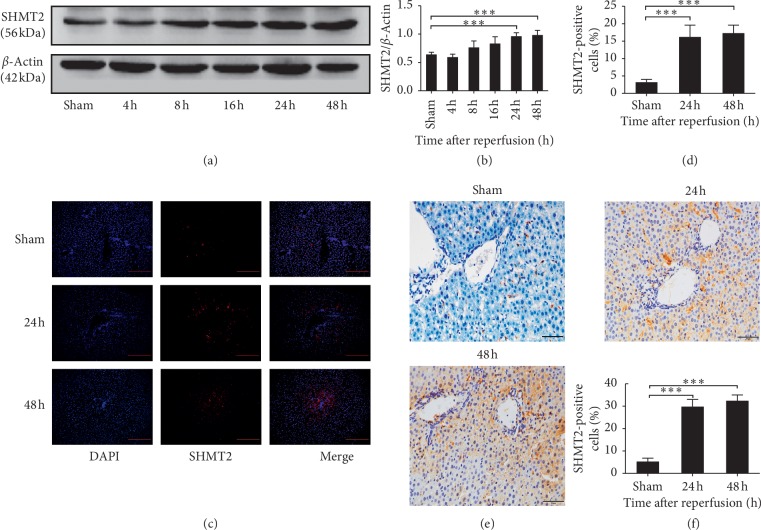
Expression of SHMT2 after IR injury in mice. (a) The expression of SHMT2 was measured by western blot analysis at 4 h, 8 h, 16 h, 24 h, and 48 h after IR. (b) Quantification of SHMT2 levels in WB. Values represent the mean ± SD of at least three independent experiments. (c) SHMT2 detected in representative sections by immunofluorescence (IF) at 24 h and 48 h after IR (magnification: 400x). (d) Quantification of SHMT2-positive cells in stained sections (^*∗∗∗*^*p* < 0.001). (e) SHMT2 detected in representative sections by IHC at 24 h and 48 h after IR (magnification: ×400). (f) Quantification of SHMT2-positive cells in stained sections (^*∗∗∗*^*p* < 0.001). Values represent the mean ± SD of at least three independent experiments.

The expression of SHMT2 in liver tissue at 4 h, 8 h, 16 h, 24 h, and 48 h after IR injury detected by WB is shown in Figure 1(a). The sham group was used as a control. Quantification of SHMT2 expression is shown in Figure 1(b). The expression level of SHMT2 gradually increased after IR injury and peaked at 24 h after IR injury (*p* < 0.001). The expression of SHMT2 detected by IF at 24 h and 48 h after IR injury is shown in Figure 1(c). The sham group was used as a control. The expression of SHMT2 was significantly elevated at 24 h and 48 h after IR injury when compared with that of the Sham group. The result is in accordance with the WB data. Quantification of the SHMT2-positive cells by IF staining (*p* < 0.001) is shown in Figure 1(d). In IHC staining, we obtained similar results as shown in Figures 1(e) and 1(f).

### 3.2. Knockdown of SHMT2 Worsens Hepatic Ischemia-Reperfusion Injury in Mice

**Figure 2:**
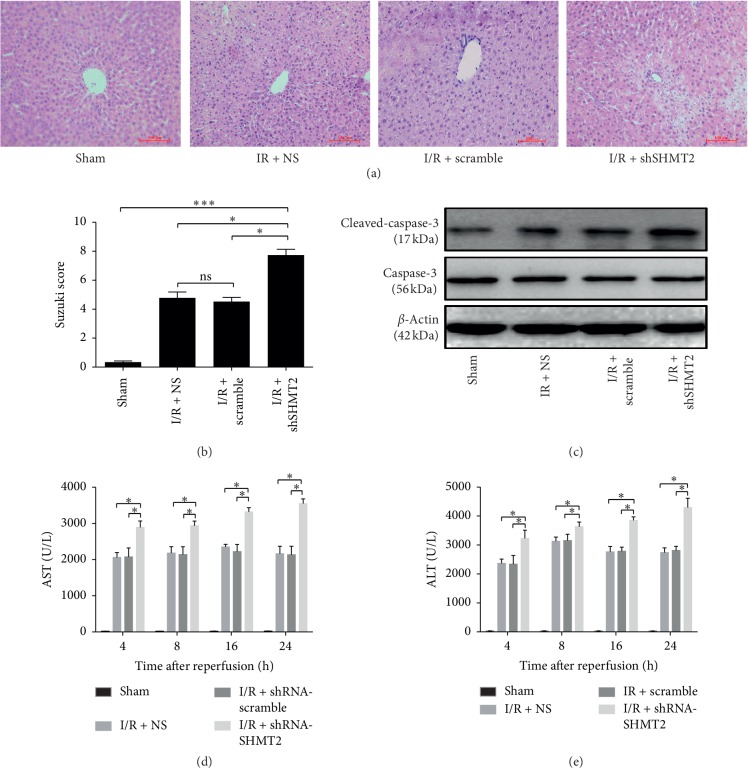
Knockdown of SHMT2 worsens hepatic IR injury in mice. (a) H&E-stained images show necrotic areas, histological damage, and liver architecture in mouse livers from NS, scramble, and shSHMT2 groups at 24 h after IR (original magnification: 400x). (b) Suzuki scores at 24 h after IR. (c) Total and cleaved protein abundances of caspase-3 in IR liver tissue from NS, scramble, and shSHMT2 groups at 24 h after IR. Serum concentrations of ALT (d) and AST (e) at 4 h, 8 h, 16 h, and 24 h after IR (*n* ≥ 4, ^*∗*^*p* < 0.05, ^*∗∗∗*^*p* < 0.001). Sham was used as a control. Values represent the mean ± SD of at least three independent experiments.

In H&E-staining images, as shown in Figure 2(a), the livers in the scramble group showed fewer necrotic areas, less histological damage, and better preservation of liver architecture than those in the shSHMT2 group. Accordingly, the Suzuki score was significantly elevated at 24 h after IRI in the shSHMT2 group compared with that of the scramble group and NS group (Figure 2(b)). Accordingly, the expression of cleaved caspase-3 at 24 h after IR was significantly elevated in the shSHMT2 group (Figure 2(c)). The serum AST levels in mice from the shSHMT2 group at 4 h, 8 h, 16 h, and 24 h after IR injury were significantly higher than those in the scramble group, and serum ALT levels in mice from the shSHMT2 group at 4 h, 8 h, 16 h, and 24 h after IR injury were significantly elevated, while there was no such difference between the scramble and NS groups (Figures 2(d) and 2(e)).

### 3.3. Knockdown of SHMT2 Promotes IR-Induced Apoptosis of Hepatocytes and Activates JNK Signaling In Vivo

**Figure 3:**
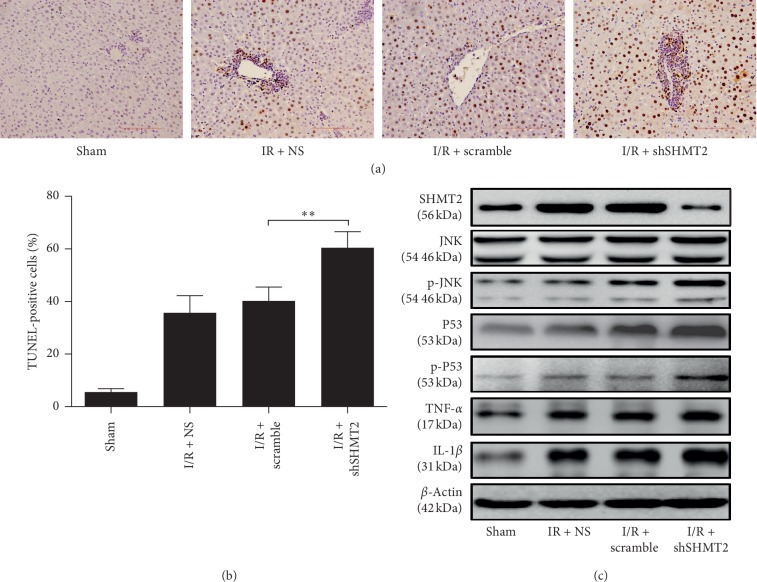
Knockdown of SHMT2 aggravates IR-induced apoptosis of hepatocytes. (a) TUNEL staining shows cellular apoptosis in mouse livers from NS, scramble, and shSHMT2 groups at 24 h after IR (original magnification: 400x). (b) Quantification of TUNEL-positive cells in NS, scramble, and shSHMT2 groups at 24 h after IR (^*∗∗*^*p* < 0.01; *n* ≥ 4). (c) Protein abundances of SHMT2, JNK, p-JNK, P53, p-P53, TNF-*α*, and IL-1 in IR liver tissue from NS, scramble, and shSHMT2 groups at 24 h after IR. Sham was used as a control. Values represent the mean ± SD of at least three independent experiments.

We tested the effect of SHMT2 interference on IRI-induced apoptosis of hepatocytes. In the representative liver sections stained by TUNEL, the shSHMT2 group had markedly higher apoptosis than that of the scramble group at 24 h after IR (Figure 3(a)). The number of TUNEL-positive cells (TUNEL-positive cells/10^3^ hepatocytes) was greater in the shSHMT2 group than in the scramble and NS groups (Figure 3(b)). We detected the expression levels of SHMT2, JNK, p-JNK, P53, p-P53, TNF-*α*, and IL-1 in liver tissue 24 h after IR injury. The expression levels of p-JNK, p-P53, TNF-*α*, and IL-1 were significantly higher in the shSHMT2 group than in the scramble and NS groups (Figure 3(c)).

### 3.4. Overexpression of SHMT2 Protects Hepatocytes against Hypoxia-Induced Apoptosis by Suppressing JNK Signaling

**Figure 4:**
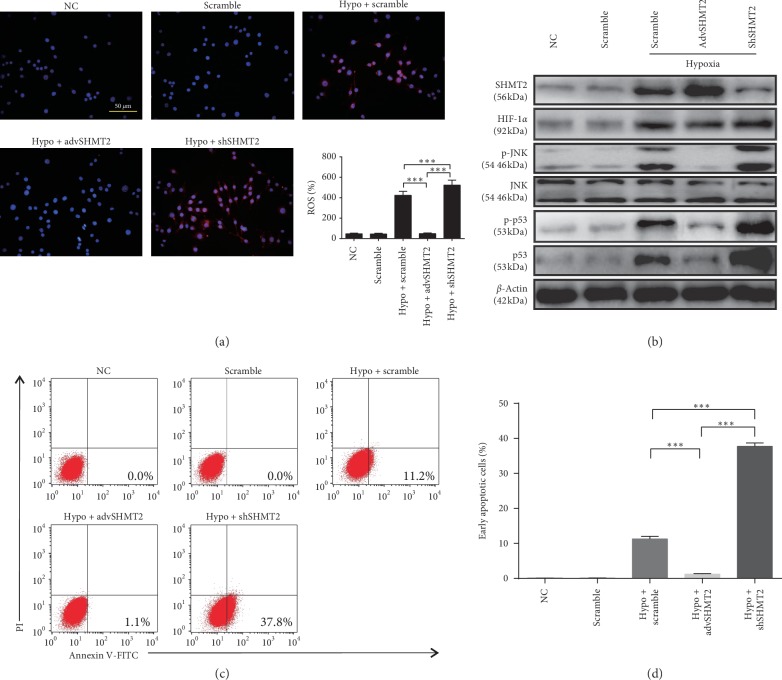
Knockdown of SHMT2 aggravates IR-induced apoptosis of hepatocytes. Intracellular generation of ROS in primary hepatocytes by transforming H2DCFDA to DCF via an oxidative reaction and quantification data for the ROS level of each group after hypoxia reoxygenation (scale bars: 50 *μ*m, *n* ≥ 4, ^*∗∗∗*^*p* < 0.001). (a) NC and scramble were used as controls. Values represent the mean ± SD of at least three independent experiments. (b) The expression of SHMT2, HIF-1*α*, p-JNK, JNK, p-p53, and p53 measured by western blot analysis after hypoxia reoxygenation. NC and scramble were used as controls. (c) The effect of SHMT2 on hypoxia-induced apoptosis in primary hepatocytes was determined using Annexin V/PI staining and flow cytometry. NC and scramble were used as controls. (d) Quantification of early apoptotic cells. Values represent the mean ± SD of at least three independent experiments.

Under hypoxia reoxygenation in vitro, the ROS production of primary hepatocytes in the scramble group was elevated but was strongly inhibited in the advSHMT2 group. Interfering with the expression of SHMT2 in primary hepatocytes resulted in ROS levels that were significantly higher than those in the scramble group (Figure 4(a)). These results indicated that the expression of SHMT2 in hepatocytes could ameliorate hypoxia-induced ROS production. HIF-1*α* is an important regulator of oxygen balance. The expression of HIF-1*α* in the hypoxia-induced scramble group and advSHMT2 group was significantly higher than that in the shSHMT2 group. Moreover, compared with expression levels in the scramble group, the expression levels of p-JNK and p-p53 pathway proteins in the advSHMT2 group were repressed, but high expression of p-JNK and p-p53 proteins was observed in the shSHMT2 group (Figure 4(b)). Additionally, the number of viable apoptotic cells was prominently decreased in the advSHMT2 group and increased in the shSHMT2 group (Figure 4(c)). Quantification of early apoptosis of cells is shown in Figure 4(d).

## 4. Discussion

Serine hydroxymethyl transferase (SHMT) is a pyridoxal-5′-phosphate-dependent enzyme functioning in the serine/glycine synthesis pathway and single-carbon metabolism, which provides essential precursors for protein and nucleic acid synthesis for cancer growth and metastasis [16]. Two types of SHMT (SHMT1 and SHMT2) genes have been discovered in the human genome [17]. In mammalian cells, SHMT1 and SHMT2 are encoded by different genes and are widely present in the cytoplasm and mitochondria, respectively. SHMT1 is mainly expressed in hepatic and renal cells, but SHMT2 is a key metabolic enzyme that catalyzes the conversion of serine to glycine and the folate cycle [18]. Most studies have confirmed that SHMT2 expression increases significantly in all types of cancer and plays a significant role in cancer cell growth and invasiveness [19–22]. SHMT2 knockout in hepatocellular carcinoma cell lines reduces cell growth and tumorigenicity in vitro and in vivo [23]. However, the specific effect of SHMT2 on IRI is still unclear. In this study, the findings suggest that SHMT2 interference worsens hepatic IRI mice. To the best of our knowledge, this is the first study to elucidate the expression profiles and impacts of SHMT2 in a mouse IRI model.

Hepatic IRI is a crucial pathological process in liver transplantation, as well as in shock and trauma [24]. Although the latest studies verified a protective effect of shikonin and tea polyphenols on hepatic IRI, there was still no effective treatment in clinical use [24, 25]. Finding a mechanism that ameliorates IRI is urgently needed. Ischemic conditions cause the depletion of adenosine triphosphate and accumulation of toxic which eventually leads to oxidative stress due to reperfusion-induced reactive oxygen intermediate production [26]. During IRI, a number of proinflammatory cytokines are released, and activated neutrophils are recruited to the liver parenchyma [27, 28]. The present data showed that the level of SHMT2 was elevated significantly after IR injury (Figures 1(a)–1(f)). This might be induced by IR-induced hypoxia.

According to histological analysis, more necrosis was observed in the shSHMT2 group (Figures 2(a) and 2(b)). The level of cleaved caspase-3 was increased in the shSHMT2 group (Figure 2(c)). The serum ALT levels in mice from the shSHMT2 group at 4 h, 8 h, 16 h, and 24 h after IR injury were significantly increased (Figures 2(d) and 2(e)). These data strongly support that SHMT2 has a protective role against liver IRI. The mechanism might lie in the fact that high expression of SHMT2 can limit pyruvate kinase isoform (PKM2), which can reduce oxygen consumption and give proliferating cells a survival advantage in poorly vascularized regions [29–31]. Moreover, previous studies showed that SHMT2 maintained a redox balance during hypoxia, and depletion of SHMT2 elevated the level of ROS, causing the death of cells under hypoxia [6]. Thus, interference with SHMT2 aggravates the apoptosis of liver cells after IRI. JNK can be activated by hepatic I/R injury. In vivo, JNK signaling was activated by IRI and promoted by SHMT2 interference (Figure 3(c)). In vitro, the production of ROS was induced by hypoxia, relieved by SHMT2 overexpression, and exacerbated by SHMT2 knockdown (Figure 4(a)). JNK signaling was activated by hypoxia, and the overexpression of SHMT2 suppressed JNK signaling (Figure 4(b)) and alleviated the apoptosis of hepatocytes (Figures 4(c) and 4(d)). Thus, overexpression of SHMT2 suppressed apoptosis in hepatocytes, and it might be related to JNK signaling.

**Figure 5:**
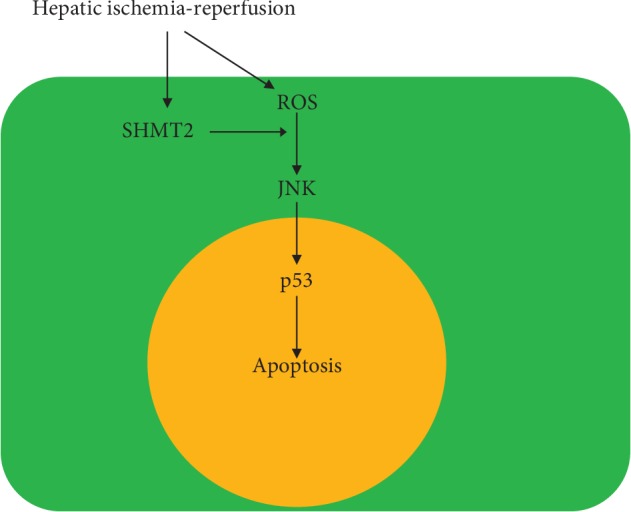
A schematic model of the upregulated ROS/JNK/P53 cell apoptosis pathway by hepatic ischemia-reperfusion injury and SHMT2 interference in the mouse liver.

In conclusion, the data of the present study revealed that the level of SHMT2 was elevated after IR injury. Interference of SHMT2 worsened IRI through the ROS/JNK/p53 signaling pathway (Figure 5). SHMT2 might be a therapeutic target to improve the prognosis of patients with end-stage liver disease who are going to receive subtotal hepatectomy and liver transplantation.

## Data Availability

The datasets used and/or analyzed during the current study are available from the corresponding author on reasonable request.

## Abbreviations

SHMT2: Serine hydroxymethyltransferase 2
IR: Ischemia-reperfusion
ALT: Alanine aminotransferase
AST: Aspartate aminotransferase
LT: Liver transplantation
PKM2: Pyruvate kinase
TNF-*α*: Tumor necrosis factor-*α*
IL-10: Interlukin-10
JNK: c-Jun NH2-terminal kinases
IVC: Inferior vena cava
HBSS: Hanks balanced salt solution
TUNEL: Terminal deoxynucleotidyl transferase dUTP nick end labeling
ROS: Reactive oxygen species
HIF-1a: Hypoxia-induced factor-1a
IF: Immunofluorescence
IRI: Ischemia-reperfusion injury
MAPKs: The mitogen-activated protein kinases.
